# Tailoring an auricular point acupressure self-management program for chronic pain: lessons learned from rural partners

**DOI:** 10.3389/fpain.2026.1851109

**Published:** 2026-09-04

**Authors:** Jungkyung Min, Jennifer Kawi, Rupsikha Bora, Sarah Pace, Tonychris Nnaka, Hulin Wu, Jane Bolin

**Affiliations:** 1 Department of Research, Cizik School of Nursing, The University of Texas Health Science Center at Houston, Cizik School of Nursing, Houston, TX, United States; 2 College of Nursing, UNT Health Fort Worth, Fort Worth, TX, United States; 3 Department of Biostatistics and Data Science, School of Public Health, The University of Texas Health Science Center at Houston, Houston, TX, United States

**Keywords:** auricular point acupressure, chronic musculoskeletal pain, rural population, community engagement, self-management, nonpharmacological treatment, program adaptation

## Abstract

**Introduction:**

Rural residents experience a disproportionate burden of chronic musculoskeletal pain and face persistent barriers to nonpharmacologic care. To address these gaps, we developed an evidence-based, app-delivered auricular point acupressure self-management (APA-SM) program and adapted it through sustained engagement with rural partners. This paper synthesizes lessons learned from rural partner engagement and describes how these insights informed APA-SM's design and refinement for rural settings.

**Methods:**

This paper summarizes lessons learned from three complementary approaches used to tailor the APA-SM program for rural settings: (1) four community advisory board meetings with 13 members across Texas and South Carolina; (2) focus group interviews with rural partners (*N* = 24; patients, providers, and administrators); and (3) a pilot feasibility study with rural adults (*N* = 11), using remote intervention training, app-based instructions, and post-participation interviews.

**Results:**

Partner engagement shifted APA-SM from a researcher-led program adaptation to a community co-design approach. Adaptations emphasized trust and workflow fit through community health workers and clinic-liaison roles, alignment with clinic priorities, and integration to reduce clinic burden. Community accessibility was enhanced through demonstrations and skills training that cultivated local champions. Participant engagement and retention were supported by plain-language, bilingual materials, low-tech laminated guides, and milestone-based incentives. These refinements improved the program's cultural relevance, feasibility, and acceptability in rural settings.

**Discussion:**

Early, reciprocal engagement with rural partners transformed APA-SM into a culturally grounded and operationally feasible program positioned for sustainable use in rural settings. The co-design principles developed here offer a transferable framework for pragmatic implementation of nonpharmacologic interventions in underserved communities.

## 1 Introduction

Chronic musculoskeletal pain (CMP) is defined as a persistent or recurrent pain condition arising from muscles, joints, or bones and lasting longer than three months (1). According to the most recent national estimates from the 2023 National Health Interview Survey, 24.3% of U.S. adults had chronic pain and 8.5% had high-impact chronic pain, defined as chronic pain that frequently limits life or work activities in the past 3 months, with the prevalence of both increasing as urbanization decreased (2). While CMP substantially impairs daily functioning and quality of life nationwide, rural populations in the United States face a disproportionate and growing burden, with prevalence rates significantly higher (28.1%) than those in urban settings (16.4%) (3, 4). This pain disparity extends beyond prevalence to access and quality of pain care. System-level constraints such as geographic isolation, limited availability of pain specialists, and scarce local resources delay guideline-concordant management (4). These systemic barriers often force a reliance on opioids, despite federal guidelines urging the use of non-pharmacologic, self-management interventions as first-line therapy (5–7). For rural and low-income Latino populations, these challenges are further compounded by underinsurance and cultural barriers, making them significantly less likely to receive non-opioid, evidence-based pain treatments (4, 7–9). While rural residents express a strong desire for sustainable, self-directed care, research gaps remain in how to effectively deliver and implement these interventions within the unique constraints of rural communities (7, 10).

To address these gaps, we developed the Auricular Point Acupressure-Self-Management (APA-SM) program. With cultural and historical origins in traditional Chinese medicine, APA is a noninvasive, needle-free approach in which small seeds or pellets secured to selected ear points provide sustained stimulation through brief, repeated pressure (11). Its potential value for rural pain management lies in its ability to support pain relief and active self-management at home, potentially reducing reliance on repeated facility-based care (12). Experimental studies, including neuroimaging and biomarker research, suggest that auricular stimulation may influence nociceptive signaling, inflammatory mediators, and pain-related neural activity, providing biologic plausibility for its potential analgesic effects (13–17). Systematic reviews and meta-analyses suggest that APA may improve short-term pain outcomes, particularly for musculoskeletal conditions (11, 18, 19). However, confidence in these findings is limited by small and heterogeneous studies, variation in treatment protocols, inconsistent evidence for functional and longer-term outcomes, and uncertainty regarding the optimal auricular points and delivery approach (18, 20, 21). A recent, fully-powered randomized controlled trial on interventionist-administered APA provided evidence of improvements in pain and physical function, sustained at 6-month follow-up (17). However, app-supported self-administration of APA needs further investigation and although self-administered APA has been shown to be feasible in small pilot studies, its effectiveness and sustainability under routine, minimally supported real-world conditions remain unclear. This evidence gap provided the rationale for investigating the adaptation of APA-SM for rural communities.

APA-SM was designed as a multi-component program that can help overcome traditional geographic barriers to pain care: a smartphone app serves as the scalable delivery platform for pain self-monitoring and other participant-facing content, while APA self-administration skills are taught through structured coaching provided by trained community health workers (CHWs). The present paper describes the community engagement carried out to refine APA-SM in preparation for a planned pragmatic randomized trial in rural settings, in which participants will be randomized to receive the coaching component either in person or remotely. Community engagement is a collaborative process of working with groups connected by geography or shared interests to address issues affecting their well-being (22–24). For underserved populations, effective engagement requires regular, bidirectional interactions to solicit input and involve community members in the dissemination of findings (25). In this project, we engaged three complementary groups to capture diverse perspectives: (i) Community Advisory Board (CAB) meetings to guide strategic priorities; (ii) focus group interviews with rural partners to align workflows and outreach channels; and (iii) a pilot study with rural adults to test feasibility and user experiences. The present paper synthesizes the lessons learned from this engagement to inform the design of the forthcoming trial and to provide a transferable framework for other researchers implementing non-pharmacologic self-management interventions in comparable settings.

## 2 Materials and methods

### 2.1 Study design

This paper presents lessons learned from a multi-phase, community-engaged co-design process used to tailor the APA-SM program for rural settings. Rather than reporting a single qualitative study, this paper summarizes stakeholder and participant insights generated across three complementary activities: (1) strategic guidance through CAB meetings, (2) focus group interviews with rural partners to inform program development, and (3) a pilot feasibility study with rural adults with CMP. This study was approved by the Institutional Review Board (IRB) of the University of Texas Health Science Center at Houston (IRB No. HSC-SN-24-0182).

### 2.2 Settings and sampling

Activities were conducted with rural partners and adults in Texas and South Carolina. Sampling was purposive and role-based rather than probability-based. Participants were sought because they could contribute one or more of the following perspectives: lived experience with chronic musculoskeletal pain; knowledge of rural clinical or community workflows; experience serving rural or Spanish-speaking populations; or direct experience using the pilot APA-SM program. Recruitment occurred through established rural clinical and community partnerships.

The CAB comprised 13 members representing a range of rural healthcare stakeholders across TX and SC, including healthcare administrators (CEOs, chief medical officers, and executive directors of rural clinics and community organizations), pain management providers (physicians, certified registered nurse anesthetists, and advanced practice registered nurses), community health worker leadership, allied health providers, and individuals representing patient and lived-experience perspectives. Because the CAB was constituted to provide institutional, professional, and lived-experience representation from rural partner organizations, individual demographic data were not systematically collected for CAB members.

### 2.3 Engagement activities and data sources

Three complementary engagement activities were conducted sequentially to inform the adaptation of APA-SM for rural settings (Table 1). The CAB provided ongoing strategic guidance and interpretation across four meetings, with members reviewing community priorities, feasibility, workflow fit, and emerging program adaptations. Focus groups conducted before pilot implementation elicited perspectives from rural patients with CMP, pain care providers, CHWs, significant others, and healthcare administrative, policy, or payer stakeholders regarding intervention acceptability, trusted outreach pathways, and clinic workflow requirements. The subsequent pilot study examined the initial delivery, feasibility and user experience of APA-SM among rural adults with chronic musculoskeletal pain, with post-participation interviews identifying usability challenges, training needs, technology-related barriers, and support requirements. Together, these activities generated complementary records that informed the iterative refinement of the APA-SM program. CAB records included summarized meeting notes and documented action items recorded by one research team member. Focus group records included audio recordings, transcripts, and analytic summaries, while pilot records included feasibility records and audio recordings and transcripts of semi-structured post-participation interviews.

**Table 1:** Overview of engagement activities, participants, and data sources.

Activity	Purpose	Participants	Primary records	Timing
Community Advisory Board	Strategic guidance and interpretation	13 members across Texas and South Carolina	Summarized meeting notes and documented action items recorded by one research team member	October 2024–July 2025; Four meetings
Focus groups	Elicit perspectives before pilot implementation	24 rural patients with CMP, pain care providers, CHWs, significant others, healthcare administrators, policymakers, payors, and staff	Audio recordings, transcripts, and analytic summaries	October–December 2024; Before pilot implementation
Pilot study	Test initial delivery, feasibility, and user experience	11 rural adults with CMP	Feasibility records; audio recordings and transcripts of semi-structured post-participation interviews	January–February 2025; 2-week intervention

### 2.4 Activity-specific procedures

#### 2.4.1 Phase 1: CAB meetings

The CAB served as a central mechanism for engaging stakeholders throughout the project. In rural health disparity research, stakeholder involvement can strengthen engagement of underserved communities and improve the relevance and acceptability of intervention strategies (26–28). A total of 13 members were recruited, representing cultural knowledge, expertise, and lived experiences relevant to CMP and rural healthcare delivery. The CAB was established in September 2024 and met four times (quarterly) over a 1-year period. CAB members received compensation for their participation, and attendance was recorded at each meeting. Because all CAB meetings were conducted remotely to accommodate members across Texas and South Carolina, food was not provided. In the first meeting in October 2024, members assessed the APA-SM intervention and provided initial feedback on feasibility, alignment with rural clinic workflows, and cultural acceptability. In the second meeting in January 2025, they recommended strategies to enhance engagement including incentives, involvement of nontraditional provider partners, and framing APA-SM as an adjunct to reduce provider burden in pain care. In the third meeting in April 2025, members reviewed APA pilot training progress at both sites, discussed recruitment and retention plans, and prioritized additional memoranda of understanding (MOUs) with rural partners. In the final meeting in July 2025, the discussion focused on community engagement approaches, including trusted local champions, concise one-page materials, and targeted outreach channels. Collectively, CAB input informed the strategic direction of the APA-SM program and guided the refinements described in the Results section.

#### 2.4.2 Phase 2: focus groups and pilot feasibility study

To further inform program refinement, two additional engagement activities were conducted sequentially under a single IRB approval: focus group interviews with rural partners and a pilot feasibility study with rural adults with CMP. The focus group activity consisted of interviews with 24 participants, recruited through rural partnerships in Texas and South Carolina, to explore perspectives on pain management and the acceptability of APA-SM. Participants represented three stakeholder groups: (1) pain care providers, community health workers, and significant others, (2) healthcare administrators (including policymakers, payors, and staff), and (3) patients with CMP. These focus groups were conducted between October and December 2024 before piloting the intervention, ensuring that findings directly informed program design.

The subsequent pilot study tested the feasibility of self-administered, app-based APA-SM among rural adults with CMP. Between January and February 2025, 11 rural adults with CMP participated in a two-week trial of the app-based APA-SM program. With step-by-step remote assistance from the study team, participants installed the APA-SM smartphone app on their personal devices and received APA kits by mail. Each kit included a probe for identifying auricular points, Vaccaria seeds or pellets secured with waterproof, latex-free tape, tweezers for seed placement when needed, and alcohol wipes to dry the ear prior to seed placement. Detailed instructional videos delivered through the APA app accompanied the APA materials, consistent with prior publications (12, 29). Semi-structured interviews were completed at the end of the pilot to gather participant feedback on feasibility, usability, and overall experience with the program. An IRB-approved letter of information was used for the focus group interviews, and written informed consent was obtained from all pilot study participants prior to enrollment. Both focus group and pilot study participants received compensation for their time.

### 2.5 Synthesis of lessons learned

Data were first reviewed within each engagement activity and then integrated across activities. CAB data consisted of summarized meeting notes and documented action items. Focus-group and post-pilot interviews were audio-recorded, transcribed, and coded using ATLAS.ti. Two team members reviewed the findings, and differences in coding or interpretation were resolved through discussion. To reduce researcher bias, the original data source was retained for each finding, and stakeholder feedback was distinguished from the research team's adaptation decisions. Findings from the three activities were then organized in a structured matrix and grouped into five domains: trust-building, partnership strategies, workflow integration, community outreach and training, and engagement and retention. Similar findings across sources were combined, while relevant source-specific findings were retained and remained traceable to their original source. No data source was assigned *a priori* priority, and no directly conflicting or substantively contradictory recommendations were identified; therefore, cross-source adjudication was not required. Because the three activities had different purposes and predetermined stopping points, we did not claim thematic saturation across the integrated data. Instead, we reviewed the complete set of available materials until no new adaptation categories emerged and confirmed that each reported lesson was traceable to its documented source.

## 3 Results

We actively incorporated feedback from rural partners, and several key lessons emerged across the three engagement activities. Attendance was recorded for all four CAB meetings, with a majority of CAB members participating across the meetings. Participant characteristics, including preferred language, are presented in Table 2. The resulting lessons were organized into five domains reflecting barriers and facilitators relevant to tailoring and delivering APA-SM in rural settings. These domains are summarized in Table 3 and further detailed below.

**Table 2:** Participant characteristics across CAB, focus groups, and pilot study.

Characteristic	CAB members (*N* = 13)	Focus group participants (*N* = 24)	Pilot study participants (*N* = 11)
Stakeholder groups represented	Policy/payer or healthcare administration (*n* = 2); community and rural health organizations (*n* = 4); healthcare and allied health providers and CHW leadership (*n* = 4); patient and lived-experience representatives (*n* = 3)	Pain care providers, CHWs, and significant others (*n* = 6); healthcare administrators, policymakers, payors, and staff (*n* = 7); patients with CMP (*n* = 11)	Adults with chronic musculoskeletal pain
Geographic distribution	Texas (*n* = 8)	Texas (*n* = 16)	Texas (*n* = 5)
	South Carolina (*n* = 5)	South Carolina (*n* = 8)	South Carolina (*n* = 6)
Gender	Not systematically collected*	Male: *n* = 8 (33.3%)	Female: *n* = 10 (90.9%)
		Female: *n* = 16 (66.7%)	Male: *n* = 1 (9.1%)
Age	Not systematically collected*	Mean: 51.8 years	Mean: 53.7 years
		Range: 23–71 years	Range: 23–69 years
Hispanic/Latino ethnicity	Not systematically collected*	*n* = 10 (41.7%)	*n* = 7 (63.6%)
Preferred language	Not systematically collected*	English: *n* = 19 (79.2%)	English: *n* = 11 (100%)
		Spanish: *n* = 5 (20.8%)	

*Because the CAB was constituted to provide institutional, professional, and lived-experience representation from rural partner organizations, individual demographic data were not systematically collected for CAB members.

**Table 3:** Lessons learned from refining the APA-SM program through academic–rural partnerships.

Domain	Strategy	Challenges identified by partners	Lessons learned
Trust-building	Engaged and empowered CHWs as key partners and intervention coaches Ensured participant comprehension of APA and shared goals Shared published results with providers and offered authorship opportunities	Low trust in external researchers Cultural disconnect between the intervention and rural values Limited understanding of APA-SM purpose and procedures	CHW-led delivery improved trust and increased participant confidence in the program Plain-language materials and teach-back reinforced comprehension and supported informed self-management Shared dissemination enhanced reciprocity and positioned partners as co-producers of knowledge
Partnership strategies	Established structured study-to-clinic liaison connections Aligned APA-SM with healthcare facility pain management priorities Secured MOUs with nontraditional community partners	Need for responsive clinic–research coordination Misalignment between research goals and clinic priorities Limited reach beyond traditional healthcare settings	Liaison-based connections improved responsiveness and site-specific adaptation Alignment with clinic goals increased organizational buy-in MOUs expanded recruitment through trusted, nontraditional partners and reached populations outside the clinical system
Workflow integration	Streamlined rural clinic workflows and division of responsibilities Flexible, site-specific referral pathways Secured data sharing and communication protocols	Clinic workload constraints and staff shortages Rigid referral processes not compatible with diverse clinic workflows Concerns about data privacy and participant confidentiality	Division of labor reduced clinic burden by concentrating research tasks within the study team Flexible referral playbook increased compatibility across diverse clinic workflows Secure digital platforms fostered institutional trust and participant confidentiality
Community outreach & training	Tailored APA training for providers and staff Community workshops and live APA demonstrations Framing APA-SM as a supplemental tool to usual care	Limited awareness of APA-SM among rural providers and residents Provider uncertainty about how to explain and endorse the intervention Unfamiliarity with the APA technique among potential participants	Training built provider confidence and facilitated the emergence of local champions Live demonstrations made APA tangible and normalized non-pharmacologic self-management Framing APA-SM as adjunctive care enhanced uptake
Engagement & retention	Provided timely, plain-language responses to participant inquiries Barrier-targeted, incremental incentives Bilingual and low-tech materials to enhance accessibility	Participant confusion during enrollment and follow-up Practical costs of participation, including travel, time, and limited internet access Language and digital literacy challenges among rural populations	Designated points of contact and timely responses maintained relational continuity with participants Milestone-based incentives offset the practical costs of participation and reinforced engagement across the study timeline Bilingual and low-tech materials promoted equitable access for participants with limited English proficiency or digital literacy

### 3.1 Trust-building

Trust was identified as a foundational requirement for implementing APA-SM in rural settings. Rural partners consistently emphasized that the success of any new intervention depends on whether it is perceived as genuinely responsive to community needs. Three interconnected strategies emerged for fostering this trust: (1) engaging and empowering community health workers (CHWs) as intervention coaches, (2) ensuring participant comprehension and shared goal-setting, and (3) establishing reciprocity with local providers through co-production of knowledge.

#### 3.1.1 Engaged and empowered CHWs as key partners and intervention coaches

Rural partners reached a consensus that CHWs are essential partners and coaches for the successful adoption of APA-SM. Partners described CHWs as trusted messengers whose shared language, cultural affinity, and social embeddedness uniquely position them to bridge the gap between an integrative intervention and community values. Guided by this input, the study team shifted the role of trained CHWs from recruiters to active intervention coaches who coached proper seed placement, provided culturally appropriate reminders, and advocated for those facing usability barriers with the app or materials. Rural partners reported that this shift was pivotal because their involvement not only increased confidence in APA-SM but also mitigated skepticism toward outside researchers by rooting the program in trusted local relationships.

#### 3.1.2 Ensured participant comprehension of APA and shared goals

Rural partners emphasized that trust in a novel, non-pharmacologic modality begins with transparent communication about the intervention itself. In response, the research team implemented a concise, plain-language orientation covering what APA is, how it may alleviate pain, expected benefits and effects, its noninvasive nature, and how it complements usual care. To verify understanding, a brief teach-back approach was used to confirm participant understanding and clarify immediate next steps. Furthermore, responding to requests for more tangible structure, we provided participants with simplified daily schedules (e.g., morning–midday–evening) and guided them to set small, measurable goals for APA use. These actions reinforced shared expectations and enhanced the transparency of the self-management process, establishing a foundation of mutual understanding between participants and the research team.

#### 3.1.3 Shared published results with providers and offered authorship opportunities

CAB members and rural providers indicated that trust between academic researchers and clinical partners must be grounded in reciprocity. Specifically, they recommended that the research team formalize mechanisms for returning study results to participating clinicians and organizations and extend opportunities for co-authorship on publications. This strategy positioned rural partners as co-producers of knowledge involved in data interpretation and dissemination, moving beyond a model in which partners serve only as data sources. In practice, we established formal protocols for sharing study findings with partner clinics and offered co-authorship opportunities to individual collaborators who contributed substantively to the research process. These actions were designed to communicate accountability, affirm the value of local contributions, and build the institutional trust necessary for sustained, long-term collaboration in rural healthcare delivery.

### 3.2 Partnership strategies

Strong partnerships were essential for advancing APA-SM in rural communities. Based on rural partner feedback, the study team implemented three key strategies to formalize these collaborations: establishing structured liaison roles, aligning program goals with facility priorities, and securing formal agreements including nontraditional partners.

#### 3.2.1 Established structured study-to-clinic liaison connections

CAB members and rural partners identified the need for a direct, structured communication channel between the study team and clinic liaisons to ensure responsiveness and reduce implementation errors. In response, we implemented a “liaison-to-liaison” approach, designating a specific study team member to connect directly with a dedicated point of contact at each rural clinic. Rural partners reported that this structured connection facilitated two-way communication and helped clinics feel supported throughout implementation. Partners also noted that having a consistent liaison allowed the study team to tailor intervention activities to the specific practice realities of each site.

#### 3.2.2 Aligned APA-SM with healthcare facility pain management priorities

Rural partners underscored the importance of aligning APA-SM with existing pain management priorities of local healthcare facilities. They advised that research partnerships should reinforce, rather than compete with, facility goals such as safer opioid stewardship and the expansion of non-pharmacologic care options. By explicitly framing APA-SM as a complementary tool that supports these clinical priorities, the program gained stronger organizational buy-in from both providers and administrators.

#### 3.2.3 Secured MOUs including nontraditional community partners

Recognizing that many rural residents rely on non-clinical networks for health information, rural partners recommended expanding outreach beyond traditional healthcare settings. Consequently, we formalized partnerships by securing MOUs with nontraditional entities, including a massage therapy facility and state-level CHW associations. These formal agreements clarified roles, established accountability, and extended the program's reach through community channels that residents already use for health information and support.

### 3.3 Workflow integration

Integrating APA-SM into rural clinics required minimizing disruption to existing operations. Based on rural partner feedback, the study team implemented three strategies to achieve this: a streamlined division of labor, flexible referral pathways, and secure communication protocols.

#### 3.3.1 Streamlined rural clinic workflows and division of responsibilities

CAB members and rural partners emphasized that APA-SM must integrate into routine operations with minimal burden to an under-resourced workforce. In response, the study team established a clear division of responsibilities designed to offload administrative tasks from clinic staff. In this model, clinic liaisons initiated referrals, while the research team managed all subsequent steps, including obtaining informed consent, delivering APA-SM orientation and skills training, administering surveys, and conducting participant follow-up. Rural partners identified this reduction in staff burden as a primary facilitator for integrating the research program into the clinic.

#### 3.3.2 Flexible, site-specific referral pathways

Feedback from rural partners indicated that a uniform referral process was incompatible with the diverse workflows of various clinical sites. To address this, the study team developed a flexible referral playbook that allowed each site to select the most compatible method, such as automated electronic health record (EHR) eligibility prompts, direct provider notifications, and liaison-initiated follow-up. To further minimize clinic involvement in logistics, the study team assumed responsibility for enrollment documentation and provided routine status updates to referring providers.

#### 3.3.3 Secured data sharing and communication protocols

CAB members and rural partners emphasized the necessity of secure, well-governed data exchange to maintain institutional trust and participant confidentiality. In response, the APA-SM program implemented fully digital and secure platforms for all administrative and clinical data, utilizing tools such as DocuSign for digital signatures and REDCap for electronic informed consent and data collection. These digital workflows streamlined the enrollment process for remote participants while maintaining encrypted communication channels for all data exchanges between the research team and rural sites.

### 3.4 Community outreach and training

Outreach and training were identified as essential components for increasing awareness and confidence in APA-SM within rural communities. Based on rural partner feedback, the study team implemented three key strategies: targeted provider training, community-based demonstrations, and the strategic framing of APA-SM as a supplemental tool.

#### 3.4.1 Tailored APA training for providers and staff

CAB members and rural partners emphasized that training should build provider confidence in APA-SM and ensure consistent, patient-facing outreach across teams. In response, the research team developed and delivered a structured curriculum specifically designed to address clinician-reported barriers, such as limited time and uncertainty about the intervention. Each session consisted of: (1) a “trial-at-a-glance” overview of the APA evidence base; (2) hands-on practice in point location and seed application using step-by-step checklists; and (3) a toolkit of plain-language scripts and teach-back prompts for patient interactions. These sessions were also instrumental in identifying local champions who demonstrated sustained commitment to the program and took ownership of its implementation within their clinics. These champions served as bridges between the research team and clinical staff, modeling the use of APA-SM and ensuring unified messaging across teams.

#### 3.4.2 Community workshops and live APA demonstrations

Rural partners recommended making APA-SM visible and tangible in trusted community venues to extend outreach beyond clinical settings. In response, the study team hosted short, interactive teach-back sessions and distributed plain-language materials at rural clinics, community health centers, health fairs, and other public events. By hosting these demonstrations where residents already gather, the program leveraged local word-of-mouth networks to normalize the use of non-pharmacologic self-management. Partners identified this community-based approach as a core mechanism for linking rural residents to the program and improving overall access to the intervention.

#### 3.4.3 Framing APA-SM as a supplemental tool to usual care

Feedback from both providers and patients suggested that APA-SM should be framed as a supplemental tool that complements, not replaces, current treatments. Following this guidance, the program was positioned as a supportive, non-invasive modality that complements existing pain management plans without interference or known interactions. This framing reassured providers and patients that participation would strengthen established care relationships without disrupting them.

### 3.5 Engagement and retention

Sustaining participation in APA-SM required a proactive approach to addressing rural-specific barriers to retention. Based on rural partner input, the study team implemented three primary strategies to promote equitable engagement: streamlined communication, milestone-based incentives, and the provision of bilingual, multi-modal materials.

#### 3.5.1 Provided timely, plain-language responses to participant inquiries

CAB members emphasized the importance of responsive, accessible communication for sustaining participant engagement. Guided by this input, we designated a single point of contact at each site responsible for participant communications. All correspondence and study materials were written in plain language with minimal technical terminology and included concise, action-oriented summaries. Standardized frequently asked questions and message templates were developed for common inquiries such as eligibility, scheduling, and app navigation. The research team provided immediate feedback and monitored inquiry and resolution status. This communication protocol was designed to support informed decision-making and reduce attrition during screening, consent, and follow-up.

#### 3.5.2 Barrier-targeted, incremental incentives

Rural partners recommended an incentive structure that directly offsets the hidden costs of participation in underserved areas, such as limited broadband access and competing work-life demands. In response, we implemented a milestone-based incentive model (4). Instead of a single end-of-study payment, participants received incremental incentives linked to key stages: baseline assessment, intervention phase, mid-point follow-ups, and completion. These incentives were designed to be practical and noncoercive, including options such as gas cards and data top-ups in low-connectivity areas, and modest completion bonuses. This tiered structure was intended to reduce the upfront burden on participants and reinforce engagement across the intervention timeline.

#### 3.5.3 Bilingual and low-tech materials to enhance accessibility

To ensure accessibility for Latino and Spanish-speaking rural populations, all core elements of the APA-SM program, including consent forms, instructional videos, scripts, and surveys, were produced in English and Spanish at a plain-language level. This effort was supported by bilingual staff and trained APA coaches who assisted with consent and troubleshooting. Additionally, recognizing that not all rural residents are comfortable with app-based tools, the team provided laminated, low-tech supplemental materials, such as step-by-step seed-application instruction cards. These adaptations ensured that technology was not a barrier to participation, promoting equitable access for participants who preferred physical guides over digital-only instructions.

## 4 Discussion

The process of engaging rural partners throughout the development and testing of APA-SM demonstrated that community insights were critical for shaping an intervention that is both acceptable and operationally feasible in rural settings. This project embraced rural partners as active co-designers whose input directly shaped program adaptations at every stage. Their perspectives revealed cultural values, trust-building dynamics, and workflow realities that would not have been visible from the research team's perspectives alone. Beyond the specific strategies that emerged, the engagement process itself functioned as a form of cultural adaptation. Each form of partnership, whether through community advisory board meetings, focus group interviews, or pilot participation, contributed to iterative refinements that made the program more responsive to rural lived experiences. The lessons that emerged from these partnerships highlight that implementation success requires shared ownership, credibility, and alignment with the priorities of the communities being served, extending well beyond program content alone. By embedding these lessons into program design, APA-SM has been positioned as a community-supported, operationally grounded strategy for chronic pain self-management in rural settings.

Five domains of adaptation emerged from this process: trust-building, partnership strategies, workflow integration, community outreach and training, and engagement and retention. Each is discussed below in relation to existing literature and implications for future implementation.

### 4.1 Trust-building

Our findings highlight that trust-building for APA-SM in rural settings required a multi-level approach that addressed the relational distance between academic researchers and rural communities. Empowering CHWs as active intervention coaches was fundamental to building trust. As community boundary spanners, CHWs bridged the social distance between external researchers and local participants (30). This aligns with evidence that engagement increases when individuals are approached by culturally concordant partners who signal genuine collaboration (31, 32). Critically, our findings extend this literature by demonstrating that for an intervention like APA, which requires participants to identify specific auricular points and apply seed placement techniques, boundary spanning must include hands-on clinical coaching, not cultural mediation alone. Furthermore, ensuring participant comprehension was important for establishing trust. Partner feedback indicated that engagement with APA depended on receiving a clear, accessible explanation of the intervention. Consistent with prior findings that study comprehension facilitates trust among underrepresented populations, our teach-back approach and simplified daily schedules both verified understanding and enabled participants to take an active role in their self-management process (31). Establishing reciprocity with clinical partners further reinforced trust. Our findings reaffirm that trust is cultivated when partners are recognized as co-producers of knowledge with active roles in the research process, and that within a Community-Based Participatory Research framework, power-sharing approaches including co-authorship and returning study results are associated with stronger relational trust (22, 33–35). CAB members noted that this relationship further communicated accountability and affirmed the value of their contributions, thereby supporting sustained collaboration (36–38). This observation is consistent with previous findings that returning results to community partners remains one of the most valued but least practiced elements of participatory research (39). Although CAB members did not participate in drafting or reviewing the present manuscript, their documented recommendations informed the lessons reported here. Beyond publication, planned dissemination includes sharing findings with rural partners through local and regional presentations and engaging CAB members and CHWs in community-facing dissemination and advocacy. Based on our experience, we recommend that reciprocity mechanisms be embedded in study protocols from the design stage. Integrating these three strategies within a single program, and refining them iteratively through partner engagement, offers a practical framework that extends current approaches to community-engaged pain management. The comprehension and reciprocity strategies are broadly transferable to other community-engaged implementation efforts, while the CHW-as-coach model is particularly suited to interventions requiring procedural skill acquisition.

### 4.2 Partnership strategies

Our findings demonstrate that the operational success of APA-SM in rural communities depended on formalized, bidirectional partnerships that aligned research activities with local practice. The liaison-to-liaison model addressed the need for responsive, structured communication in resource-limited clinical settings. This structured communication model aligns with evidence that dedicated coordination roles between research teams and clinical sites reduce implementation errors and promote shared ownership of the intervention (30, 40). This structured coordination was particularly important during remote delivery across multiple sites. Consistent liaison contact helped address provider questions in real time and maintained responsiveness to each clinic's evolving needs. The sustainability of these partnerships was further reinforced by aligning the intervention with existing facility priorities. Our findings are consistent with prior research that clinical providers and administrators are more likely to endorse programs perceived as goal-concordant resources rather than external research burdens (41, 42). For APA-SM specifically, this alignment was important because the intervention introduces a modality unfamiliar to most rural providers. Framing it around priorities to which facilities were already committed helped reduce the perceived risk of adoption and positioned the program as a practical contribution to existing care goals.

Finally, the formalization of partnerships through MOUs with nontraditional entities extended the program's reach to individuals who may not routinely access clinical care, leveraging the most influential outreach mechanism in rural communities (43, 44). These nontraditional partners served as entry points for populations that traditional clinic-based recruitment would not have reached (34, 45). For interventions targeting chronic pain in rural communities, where care-seeking behavior is often shaped by factors outside the clinical system, such partnerships are not supplementary but foundational to equitable access (46).

### 4.3 Workflow integration

Our findings demonstrate that successful integration of APA-SM in rural clinics depended on designing study processes that functioned within existing clinical operations. The division of labor, in which clinic staff were responsible only for the initial referral while the research team managed all subsequent activities, directly addressed a well-documented implementation barrier in rural settings: insufficient staff capacity to absorb research-related tasks (47). This approach is consistent with evidence that clinicians are more likely to support interventions that do not increase their workload or compromise time spent on direct patient care (42). For APA-SM specifically, this workflow was important because the intervention involves multiple procedural steps, including orientation, seed placement training, app setup, and follow-up, that would be impractical for understaffed rural clinics to manage independently. By concentrating these tasks within the research team, we preserved the clinic's primary function while maintaining fidelity to the intervention protocol. The flexible referral playbook reflected a practical recognition that rural clinics operate under diverse workflows. We offered each site a menu of compatible options instead of imposing a standardized referral protocol. Consistent with this approach, using streamlined workflows with minimal-friction referral steps directly addressed known implementation barriers, such as time-consuming procedures and insufficient staff support. This site-specific approach aligns with implementation science literature indicating that adaptable interventions achieve higher adoption rates than rigid protocols, particularly in resource-limited settings (48, 49). Furthermore, the implementation of secure digital platforms for data sharing and informed consent addressed partner concerns about institutional trust and participant confidentiality. In a multisite program relying heavily on remote communication, the integrity of data infrastructure directly affects partners' willingness to participate. Our use of encrypted platforms such as REDCap and DocuSign ensured that research activities remained compliant with institutional privacy standards while enabling efficient multi-site coordination (50). These safeguards were essential for harmonizing research activities with routine clinical privacy standards, ensuring that the communication infrastructure remained both scalable and compliant within the existing healthcare system (51–53).

### 4.4 Community outreach and training

Our findings indicate that successful dissemination of APA-SM among rural communities required coordinated strategies that built provider confidence, community familiarity, and alignment with existing care expectations. Tailored provider training addressed clinician-reported barriers, specifically limited time and unfamiliarity with APA, by providing a structured, concise curriculum that could be absorbed within the constraints of busy rural practice (53, 54). This is consistent with evidence that patients regard their primary providers as the most authoritative sources of health information, making provider confidence a prerequisite for patient engagement, particularly among populations underrepresented in research (39, 55). For APA-SM, where providers must be able to explain an unfamiliar modality and respond to patient questions about auricular point stimulation, tailored training was essential to equip rural providers with the specific communication skills needed to endorse the program credibly (39). The training sessions also helped identify local champions. Continued engagement in training activities highlighted clinicians who demonstrated the commitment, credibility, and influence needed to support and promote the intervention within their teams. This aligns with a previous study suggesting that effective champions often emerge through active engagement and demonstrated capacity within their clinical settings (56). Furthermore, community-based workshops and live APA demonstrations extended the program's visibility beyond clinical settings into venues where rural residents already gather. This strategy reflects evidence that community-based visibility is a core component for raising awareness and reducing the stigma associated with non-pharmacologic pain care (57). For APA-SM, live demonstrations were particularly valuable because partner feedback indicated that hesitation stemmed primarily from unfamiliarity with the technique itself, not from resistance to complementary approaches. By making APA tangible and accessible in trusted community spaces, these workshops functioned as a recruitment pathway that clinic-based outreach alone could not have provided. Framing APA-SM as a supplemental tool within usual care, rather than a replacement, proved essential for both provider endorsement and patient acceptance. Our findings are consistent with evidence that rural patients are more receptive to complementary modalities when they are presented as adjunctive options that do not interfere with established treatments (46, 57). This approach also mirrors national clinical practice guidelines, which recommend non-pharmacologic modalities as first-line or adjunctive strategies within a shared decision-making framework (58). For APA-SM, this positioning was particularly important because the intervention asks patients to adopt a self-management practice outside their usual care setting. Framing it as complementary to their existing pain management plan reduced perceived risk and lowered the threshold for initial engagement.

### 4.5 Engagement and retention

Our findings suggest that sustaining engagement and retention in the APA-SM program required removing the practical barriers that can lead to attrition among rural participants. For chronic pain populations, engagement is deeply influenced by the quality of the relationship with the research team (59). By providing consistent, jargon-free responses and maintaining a single point of contact, we fostered a relational continuity that supported participants throughout the study timeline (60, 61). This was particularly important for APA-SM because the intervention is delivered remotely and requires ongoing self-management (61). Without regular, accessible communication, participants who encountered difficulties with the app or seed placement techniques had no immediate clinical support to turn to, creating a risk of disengagement despite continued enrollment. The milestone-based, barrier-targeted incentive model addressed what partners described as the hidden costs of participation, including time, travel, and data usage, that often erode sustained effort in rural settings (7). Unlike traditional lump-sum payments, this incremental structure provided reinforcement at each stage of the study. For APA-SM, which requires daily self-management over a multi-week period, the milestone structure was designed to maintain motivation throughout the intervention phase, not solely at completion. Furthermore, the provision of bilingual materials and low-tech alternatives addressed the persistent digital health inequities faced by Latino and rural populations. According to a recent scoping review, Spanish speakers in the U.S. often encounter significant barriers to digital health participation due to linguistic mismatches and low health literacy, which can lead to disengagement even among formally enrolled participants (61). By producing all core materials in English and Spanish, supplementing the app with laminated instruction cards, and staffing bilingual APA coaches, we created a hybrid engagement infrastructure that combined digital delivery with personalized, culturally concordant support. This approach reflects growing evidence that effective digital health interventions in underserved communities require parallel non-digital pathways to ensure equitable access (60, 61). For APA-SM specifically, this hybrid model was essential because the intervention relies on an app-based delivery system that would otherwise exclude participants with limited digital literacy or inconsistent internet access (61).

### 4.6 Limitations

This study has several limitations that should be considered when interpreting the findings. First, the small sample size in the pilot feasibility study (*N* = 11) and focus groups (*N* = 24) limits the generalizability of the findings to all rural contexts. The two study sites in Texas and South Carolina represent specific geographic and demographic characteristics that may not reflect the experiences of rural communities in other regions, such as Appalachia or the rural Midwest. Second, the findings should be interpreted as lessons related to program refinement, feasibility, and acceptability rather than as evidence of the clinical effectiveness of APA-SM. Third, participants and partners who volunteered for this study may have been more receptive to complementary approaches than the broader rural population, introducing potential selection bias. Similarly, CAB members who were engaged throughout the research process may have provided more favorable assessments of the program, raising the possibility of social desirability bias. Fourth, the two-week intervention period was sufficient to assess initial feasibility and user experience but does not capture the sustainability of APA-SM self-management behaviors over time. Fifth, while we utilized digital tools to bridge geographic gaps and provided low-tech alternatives, the persistent digital divide in rural areas remains a challenge that supplemental materials can only partially address. The extent to which app-based delivery limits participation among individuals with low digital literacy or inconsistent internet access requires further investigation. Future research should address these gaps through larger, controlled trials with longitudinal follow-up to evaluate whether community-tailored APA-SM leads to sustained pain reduction and decreased reliance on opioid medications. Studies should also examine the transferability of these co-design strategies to other rural regions and to other non-pharmacologic interventions that require procedural skill acquisition. Additionally, research is needed to assess the long-term sustainability of CHW-as-coach models and the organizational conditions under which local champions can be maintained beyond a single study period. Sixth, although focus group and post-pilot interview transcripts were analyzed using a structured coding process, CAB data were derived from summarized meeting notes and action items rather than verbatim transcripts. The integrated synthesis was intended to identify actionable lessons across complementary engagement activities rather than to generate themes using a single qualitative methodology, which may have limited the depth and consistency of cross-source interpretation. Seventh, individual demographic information was not systematically collected for CAB members. Although Table 2 reports all available participant characteristics and stakeholder roles, the absence of CAB demographic data limited our ability to assess how representative the engaged stakeholders were of the broader rural populations served.

## 5 Conclusion

Sustained engagement with rural partners transformed APA-SM from a researcher-led intervention into a community co-designed program grounded in the priorities and realities of rural practice. Through iterative refinement across five domains — trust-building, partnership strategies, workflow integration, community outreach and training, and engagement and retention, the program achieved stronger cultural relevance, operational feasibility, and acceptability in rural settings. Key adaptations included empowering CHWs as intervention coaches, aligning the program with facility pain management priorities, minimizing clinic burden through streamlined workflows, building community familiarity through live demonstrations, and removing practical barriers to participation through bilingual materials and targeted incentives. These co-design principles offer a transferable framework for researchers and clinicians seeking to implement non-pharmacologic, self-management interventions in underserved rural communities. Early, reciprocal partnerships with rural stakeholders are not an optional enhancement but a prerequisite for interventions that aim to be both effective and equitable.

## Data Availability

The de-identified data supporting the conclusions of this article, along with associated documentation (e.g., the data dictionary and metadata), will be made available through the HEAL Data Platform in accordance with the NIH HEAL Initiative Public Access and Data Sharing Policy.
